# *Cryptosporidium* secreted proteins form a complex layered interface with the host cell

**DOI:** 10.1371/journal.ppat.1014212

**Published:** 2026-05-14

**Authors:** Annet Puthenpurackal, Sandra Moreno Sanchez, Tifany Schaer, Léna Falconnet, Chengyue Niu, Oscar Vadas, Amandine Guérin

**Affiliations:** Department of Microbiology and Molecular Medicine, Faculty of Medicine, University of Geneva, Geneva, Switzerland; University of Dundee, UNITED KINGDOM OF GREAT BRITAIN AND NORTHERN IRELAND

## Abstract

*Cryptosporidium parvum*, one of the leading causes of diarrheal death in children, remodels its infection site through secreted effector proteins. Several of them accumulate at the host-parasite interface, forming a complex structure whose function and importance for infection remain poorly understood. Here, we localized and functionally characterized the putative dense granule protein DG8. We confirmed the protein to be in the dense granules, forming a ring structure once secreted at the host-parasite interface. Deletion of DG8 showed reduced infection in mice *in vivo* and early defect *in vitro*, revealing the importance of DG8 for parasite fitness. Additional secreted proteins were identified as potential partners, among which SG4, a small granule protein, was shown to partially co-localize with DG8. Applying ultrastructure expansion microscopy on intestinal sections with newly generated antibodies against DG8 and SG4, we could precisely position the ring structure above the electron-dense band, around the feeder organelle at the base of the parasitophorous vacuole membrane with an unprecedent resolution. Altogether, better visualization and understanding of the host-parasite interface through expanded intestinal tissue and effectors characterization reveal a complex and essential layered host-parasite interface.

## Author summary

The parasite *Cryptosporidium* infects and replicates within intestinal epithelial cells leading to cryptosporidiosis, a diarrheal disease. The parasite contains specialized organelles, dense and small granules, which secrete their content during invasion to various locations including the parasitophorous vacuole, a protective niche in which the parasite replicates, and the interface with the host cell. In our study, we investigated the dense granule protein DG8 and localized it forming a ring at the host-parasite interface. Although DG8 is not essential for infection, its depletion affects the parasite fitness both *in vitro* and *in vivo*. Using various pull-down experiments, we showed that DG8 is part of a larger complex of secreted proteins, including the small granules protein SG4. By expanding mouse intestinal sections and producing antibodies against DG8 and SG4, we were able to improve the resolution of the interface, enabling us to precisely localize both effectors. This approach revealed that the interface is organized in distinct layers formed by multiple secreted proteins, providing new insights into how the host-parasite interface is formed during *Cryptosporidium* infection.

## Introduction

*Cryptosporidium hominis* and *Cryptosporidium parvum* are protozoan parasites responsible for the majority of human cryptosporidiosis, an illness that can be life-threatening in immunocompromised patients and children [1]. In fact, each year an estimated 200,000 children die from acute diarrheal disease and malnutrition caused by this apicomplexan parasite [2,3]. Despite a prevalence of up to 65% in developing countries, cryptosporidiosis receives little attention from the World Health Organization and has yet to be classified as a neglected tropical disease [4,5]. The burden of infection is also evident in industrialized countries, where outbreaks primarily affect children under the age of four and are often associated with contaminated water sources (ECDC, 2024). Treatment remains a major challenge due to lack of effective drugs and the only approved therapeutic, nitazoxanide, shows reduced efficacy in immunodeficient patients and children, the population most in need [6,7]. A deeper understanding of *Cryptosporidium* pathogenesis is therefore essential for developing new drugs and vaccine candidates.

**Fig 1:**
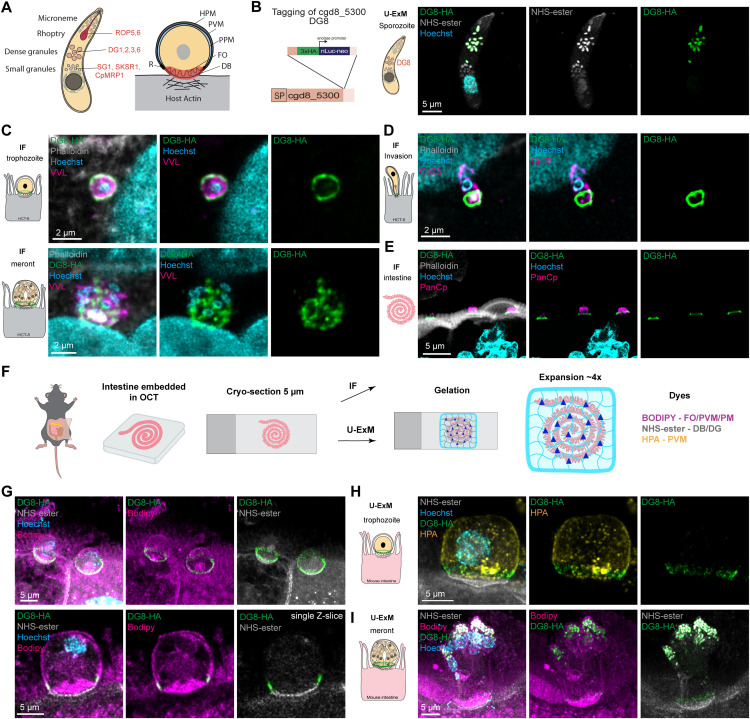
The dense granule protein cgd8_5300 accumulates as a ring structure at the interface. **(A)** Schematization of known proteins, with their organellar origin, accumulating at the interface adapted from Guérin and Striepen, 2020. DB, Dense Band; FO, Feeder Organelle; HPM, Host Plasma Membrane; PVM, Parasitophorous Vacuole Membrane; PPM, Parasite Plasma Membrane. **(B)** Left, Schematization of the tagging strategy for cgd8_5300 with the insertion of a triple hemagglutinin (HA) tag followed by a nanoluciferase and neomycin cassette. Right, U-ExM of excysted sporozoite on poly-L-lysine. NHS-ester, in grey, labels the outline of the parasite as well as the single rhoptry and the dense and small granules while Hoechst, in cyan, labels the nucleus. The HA staining in green recognizes cgd8_5300 (DG8) in the dense granules colocalizing with the large NHS-ester positive granules. **(C)** IF of DG8-HA showing an accumulation as a ring at the interface with the host cell in trophozoite (top) and in the newly formed organelles in meronts (bottom). The parasite vacuole is labelled with VVL (Vicia Villosa Lectin) in magenta, the host filamentous actin with phalloidin in grey, Hoechst is in cyan. **(D)** IF showing a sporozoite invading HCT-8 cells, labelled in magenta for the parasite pellicle by an antibody against Cp23,. Phalloidin is used to confirm the invasion by detecting the host actin polymerization dot. The HA staining in green is labelling DG8 being secreted at the infection site. **(E)** IF in intestinal tissue section of mice infected with DG8-HA. PanCp in magenta labels the parasite, phalloidin in grey the host F-actin and Hoechst the host and parasite nuclei. The HA in green showed a ring structure at the interface from a side view similar to the localization seen *in vitro*. **(F)** Schematization of the procedure to image intestine from mice infected with *Cryptosporidium* by IF or U-ExM. **(G)** U-ExM on intestinal section using BODIPY in magenta to label the lipids (HPM, PPM, PV, FO) as well as NHS-ester in grey to label structure enriched in free amine like the dense band. DG8 labels the interface as a ring above the dense band in trophozoite as seen on projection (top) and single Z- slice (bottom). **(H)** U-ExM on intestinal section using HPA in yellow as a vacuole marker as well as NHS-ester in grey to label structure enriched in free amine like the dense band. DG8 labels the interface as a ring above the dense band in trophozoite at the base of the parasitophorous vacuole. **(I)** U-ExM on intestinal section using BODIPY in magenta to label the lipids (HPM, PPM, PV, FO) as well as NHS-ester in grey to label structure enriched in free amine like the dense band and the dense granules in meronts. DG8 is visible in the dense granules as well as at the interface.

The pathogenesis of *Cryptosporidium* begins with the ingestion of oocysts, the environmentally resistant form of the parasite [8]. In humans, ingestion of as few as 10 oocysts is sufficient to cause disease [9]. Following excystation in the small intestine, the released sporozoites invade enterocytes and establish an intracellular niche known as the parasitophorous vacuole (PV) in which it replicates [8]. Unlike other apicomplexan parasites, *Cryptosporidium* remains extra-cytoplasmic, creating a unique host-parasite interface that separates it from the host cytoplasm [10]. Distinctive features of this infection site include an invagination of membrane called the feeder organelle (FO) thought to mediate nutrient acquisition, an electron-dense band (DB) composed of unknown parasite proteins, an electron dense ring-shape structure where the parasite plasma membrane (PPM) and the parasitophorous vacuole membrane (PVM) fused and a host actin pedestal which has been shown to be essential for invasion [8] (Fig 1A). The single PVM is surrounded by the host plasma membrane (HPM) and the two have been in the past referred together as a two-layered parasitophorous envelope. These observations are mostly based on electron microscopy sections that lack a 3-dimensional resolution making membranes tracking challenging, a gap that could be solved with newest techniques [11]. Despite these hallmarks, the molecular composition and the function of this interface remain poorly understood, particularly the mechanisms by which the parasite induces these modifications.

Effector proteins in apicomplexan parasites are housed within specialized secretory organelles, including micronemes, rhoptries (ROP) and dense granules (DG). In the model organism *Toxoplasma gondii*, their functions are well characterized. Micronemes secrete adhesins and motility factors to drive host cell attachment and invasion [12]. Rhoptries inject proteins in the host to build the moving junction and the PVM to subvert host cellular functions [13]. Dense granules are essential to form the PV and further manipulate host cell functions [14,15]. Collectively, secretory organelles release proteins at defined stages of *Toxoplasma’s* life cycle to mediate attachment, invasion and host cell manipulation. Although conserved, the secretory organelles of *Cryptosporidium* remain far less understood. In addition to the micronemes, the single rhoptry and the dense granules, *Cryptosporidium* features a fourth set of secretory organelles termed small granules (SG) [16]. These small vesicles (120nm diameter versus 200nm diameter for DGs), localize near the nucleus and display unique biogenesis and secretion timings compared to the other organelles [16,17]. The proteome of *Cryptosporidium* sporozoite has only been recently defined through hyperplexed localization of organelle proteins by isotope tagging (hyperLOPIT) which assigned 154 secretory proteins to these four secretory organelles [16]. Despite this advance, the function of the majority of secreted *Cryptosporidium* proteins remains unknown.

Several effectors have been detected within the PV of *Cryptosporidium*-infected cells, including the proteins DG4 and DG5 from the dense granules, SG2 from the small granules, ROP2, ROP4 and ROP7 from the rhoptry [16,18,19]. In addition, a large number of *Cryptosporidium* effectors accumulate at the host-parasite interface, but their precise functions remain unknown (Fig 1A). Among these, the two rhoptry proteins, ROP5 and ROP6, as well as the dense granules proteins, DG1, DG2 and DG3, have been reported to form a ring at the host-parasite interface [16,18]. Similarly, the small granule effectors SG1 and SKSR1 have also been detected at the interface [16,17]. Recently, the dense granule protein DG6, accumulating at the interface, as well as the SG-derived transporter CpMRP1, likely localizing at the FO, were shown to be important for parasite growth *in vivo* supporting an important role for this interface [19,20]. Collectively, these findings indicate that *Cryptosporidium* effectors from distinct secretory organelles are secreted and accumulate at the host-parasite interface, however, due to the limited availability of specific markers and antibodies, the precise location of effectors within structures and their functional significance for parasite survival remain unresolved.

In this study, we investigated the protein cgd8_5300, predicted to localize to the dense granules by hyperLOPIT. We validated its localization and examined its dynamics during infection. Cgd8_5300 was delivered to the host-parasite interface throughout the asexual cycle, where it formed a ring structure above the DB at the base of the PVM. Functional analyses revealed that parasites lacking this DG protein remained viable, but displayed reduced fitness, with a defect apparent early during infection. Pull-down experiments to identify potential partners revealed the enrichment of proteins predicted to be secreted from multiple secretory organelles. Among these, the small granule protein cgd6_3920 was tagged and localized to the host-parasite interface, where it formed a ring structure that partially overlapped with cgd8_5300. By generating antibodies against both proteins and applying ultrastructure expansion microscopy on mouse tissue infected with various HA-tag strains, we established powerful tools to visualize the complex organization of the host-parasite interface, revealing that secreted proteins occupy discrete and well-defined regions within this structure.

## Results

### Cgd8_5300 (DG8) is secreted from the dense granules to the host-parasite interface

To investigate the molecular composition of the host-parasite interface, candidate proteins were identified based on the presence of a signal peptide, high predicted intrinsic disordered regions, elevated RNA transcript levels, and the presence in the dense granules cluster in the hyperLOPIT dataset [16,21,22] (S1A Fig). Among these, cgd8_5300 was selected and endogenously tagged at its C-terminus with a triple hemagglutinin (HA) tag followed by a nanoluciferase and neomycin cassette using Cas9 mediated integration, which was verified by PCR (Figs 1B and S1B). Ultrastructure expansion microscopy (U-ExM) of excysted sporozoites revealed localization of cgd8_5300 to large N-Hydroxysuccinimide (NHS)-ester positive granules, confirming its presence in the dense granules [16] (Figs 1B and S1C). This protein was therefore named DG8 according to current nomenclature [16,19,23]. During early infection of HCT-8 human adenocarcinoma cells, DG8 formed a distinct ring at the host-parasite interface and remained present throughout the asexual replication (Fig 1C). At later stages in mature meronts, DG8 was detected in newly formed dense granule organelles, consistent with the biogenesis of previously identified dense granules proteins [16] (Fig 1C). Interestingly, the ring localization was already observed when DG8 is secreted (Fig 1D), suggesting that this structure is formed early during invasion.

### U-ExM on intestinal section reveals DG8 localization above the dense band

The *in vivo* localization of DG8 was then examined by harvesting the small intestine of infected mice, embedding it in Optimal Cutting Temperature compound (OCT), preparing 5 µm cryosections and performing immunofluorescence (IF) (Fig 1E and 1F). This approach not only enabled visualization of the parasite and DG8 under physiological conditions but also provided side views of the interface. From the side view, DG8 appeared as a line between the parasite and the host brush border, corresponding to the ring structure observed from the top view (Fig 1E). To position DG8 more precisely at the interface, we combined intestinal cryosections with U-ExM (Fig 1F). This method was paired with a panel of dyes: BODIPY to highlight lipid-rich membranes (PVM, PPM, HPM, FO), NHS-ester to label protein-rich structures (DB, DG), and Helix pomatia agglutinin (HPA) to mark the PVM. Using these markers in combination with the DG8-HA strain, we accurately observed that DG8 localized above the DB, was excluded from the FO and colocalized with the PVM in a ring staining (Figs 1G top, 1H, S1D top, and S1E). Single Z-sections provided clear spatial resolution, showing DG8 positioned above the DB within the lipid-rich region that encompasses the PVM, HPM, and PPM (Figs 1G bottom, and S1D bottom). Within the same section, fully developed meronts were visible, with BODIPY marking the PPM of individual merozoites, while NHS-ester labeled newly formed dense granules and the DB. In these stages, DG8 was largely detected within the dense granules, though a fraction remained visible at the interface, consistent with the IF results (Figs 1I and S1F).

### DG8 is important for parasite growth *in vivo* and *in vitro*

**Fig 2:**
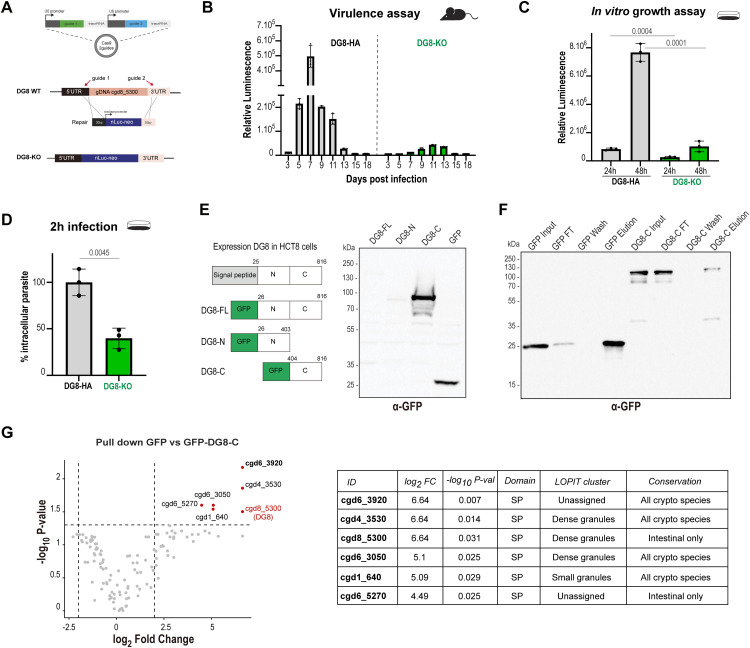
DG8 is a virulence factor important for parasite growth *in vitro* and *in vivo.* **(A)** Schematization of the knockout strategy for DG8 with the insertion of a nanoluciferase and neomycin cassette instead of the entire coding sequence of DG8 using an adapted vector containing two consecutive guides. **(B)** Representative graphic of a virulence assay showing the luminescence in the feces of 3 mice infected with either 10,000 oocysts of the DG8-HA strain or the DG8-KO strain from an equivalent passage. Parasites lacking DG8 are less virulent than the HA version (Area under the curve = 9.5-fold decrease in this experiment; 11-fold decrease in average in 3 independent experiments). 3 mice per condition in 3 independent experiments. **(C)** *In vitro* growth assay in P96 wells plate shows a decrease in growth of the mutant versus the tagged version of DG8 by 3.2 time at 24h and 7.5 time at 48h suggesting that DG8 is important for growth *in vitro.* Unpaired t test. **(D)** Infection for 2 hours *in vitro* of HA versus KO version of DG8 shows a reduction of 60% of the number of intracellular parasites in the absence of DG8. Quantified using VVL for 40 independent fields of view and normalize to 100% to the control. n = 3. Unpaired t test. **(E)** Left, schematization of the truncated versions used to express DG8 in HCT-8 cells fused to GFP. Right, Western blot using an anti-GFP on HCT-8 cells transiently transfected with GFP-DG8-FL, GFP-DG8-N, GFP-DG8-C or GFP alone. Only GFP-DG8-C-terminal shows a strong detection among the 3 versions tested. **(F)** Immunoprecipitation using GFP-trap beads on GFP or GFP-DG8-C expressing HEK293T cells and incubated with a lysate of HCT-8 infected with *Cryptosporidium*. Anti-GFP was used to reveal the membrane and detect GFP or GFP-DG8-C in the elution lines. **(G)** Volcano plot showing in red the proteins identified with at least a 2-fold enrichment and a p-value < 0.05. The full length DG8 was detected in the GFP-DG8-C alongside 5 proteins containing a signal peptide summarized in the table.

To investigate the function of DG8, a knockout mutant strain was generated. Due to recent evidence indicating polycistronic transcription in *Cryptosporidium* [24], the typical strategy of disrupting only the coding sequence was adapted to remove the entire DG8 locus. To achieve this large-scale deletion, an updated Cas9/guide plasmid encoding two consecutive guide RNAs with a single Cas9 was engineered, enabling simultaneous cleavage at the 5′ and 3′ UTRs (Figs 2A and S1G). The use of a single plasmid ensured co-delivery of both guides, thereby increasing the likelihood of complete gene excision. DG8-depleted parasites were recovered, and successful cassette integration as well as complete absence of DG8 genomic DNA were confirmed by PCR (S1G Fig). During mutant generation, we noticed reduced luciferase activity and low parasite recovery after purification, suggesting a possible growth defect in mice. To validate this, mice were infected with equal doses of DG8-HA or DG8-KO parasite strains, and luciferase activity was monitored in fecal samples until parasite clearance (Figs 2B and S1H). As expected, DG8-HA parasites exhibited a peak of infection followed by clearance within three weeks. In contrast, DG8-KO parasites displayed an average 11-fold reduction in infection burden across three replicates with a lower peak of infection indicating a role for DG8 in parasite virulence. We next tested whether this phenotype extended to *in vitro* growth in HCT-8 cells. In 96-well plates *in vitro* growth assays, DG8-KO parasites showed reduced luminescence compared with DG8-HA parasites, with a 3.2-fold reduction at 24 hours that reached 7.5-fold at 48 hours (Figs 2C and S1I). Given that DG8 is secreted early during infection, we quantified intracellular parasite numbers after 2 hours of HCT-8 infection. The KO strain already exhibited a significant defect at this stage with 60% less intracellular parasites (Fig 2D), suggesting that DG8 contributes to the establishment of infection, likely through proper formation of the host-parasite interface. Additional U-ExM experiments were performed to observe the formation of the interface during invasion with NHS-ester without obvious defect except a reduced number of invading parasites in the KO strain (S1L Fig).

### Pull down reveals parasite partners of DG8

To further explore DG8 function, we sought to identify its interacting partners. We first attempted endogenous tagging of DG8 with miniTurbo to capture proteins in proximity upon addition of biotin [25]. The tagging was successful, however, microscopy revealed defective secretion (S1J and S1K Fig), suggesting that the bulky tag disrupted DG8 trafficking or secretion, consistent with previous reports on secreted proteins [26]. As an alternative approach, DG8 fused to GFP was over-expressed in a heterologous system to be used as a bait in pull down experiments, thereby avoiding the need for large numbers of transgenic parasites. When GFP was fused to the N-terminus of the full-length recodonized DG8 lacking its signal peptide (GFP-DG8-FL), no protein bands were detected by western blot with anti-GFP (Fig 2E). Optimization attempts, including replacing polyethylenimine with lipofectamine transfection reagents and switching from HCT-8 to highly transfectable HEK-293T cells, still failed to yield robust expression of GFP-DG8-FL. Truncation experiments showed that only the C-terminal portion of DG8 (GFP-DG8-C) was stably expressed and detectable by western blot (Fig 2E). Therefore GFP-DG8-C was expressed, captured onto GFP-trap beads, and incubated with lysates from HCT-8 cells infected with wild-type parasites for 3 hours, a time point at which the host-parasite interface is established (Fig 2F). Compared with the GFP control, the GFP-DG8-C condition was enriched for the endogenous full-length DG8 as well as five additional proteins containing signal peptides (Fig 2G and S1 Table). Based on the hyperLOPIT dataset, two of the identified proteins are predicted to be dense granule proteins (cgd4_3530, cgd6_3050), one to be a small granule protein (cgd1_640), and two were detected but remained unassigned (cgd6_3920, cgd6_5270). Revaluation of available datasets on CryptoDB suggested that cgd6_3920 is possibly a small granule protein, while cgd6_5270 resembles a rhoptry protein (S2A Fig). Altogether, these findings suggest that DG8 forms part of a protein complex of secreted proteins originating from several organelles at the host–parasite interface, whose exact composition remains to be characterized.

### Cgd6_3920 is a small granule protein (SG4) that accumulates at the interface

**Fig 3:**
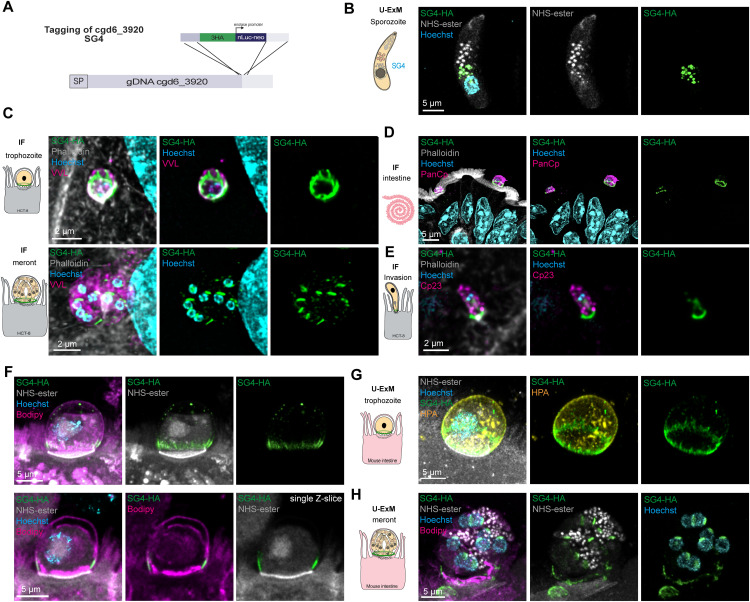
Cgd6_3920 is a small granule protein accumulating at the interface. **(A)** Schematization of the tagging strategy for cgd6_3920 with the insertion of a triple hemagglutinin (HA) tag followed by a nanoluciferase and neomycin cassette. **(B)** U-ExM of excysted sporozoite on poly-L-lysine. NHS-ester, in grey, labels the outline of the parasite as well as the single rhoptry and the dense and small granules while Hoechst, in cyan, labels the nucleus. The HA staining in green recognizes cgd6_3920 (SG4) within the small granules with its peri-nuclear localization. **(C)** IF for SG4-HA showing an accumulation as a ring at the interface with the host cell in trophozoite (top) and in the newly formed organelles in meronts (bottom). The parasite vacuole is labelled with VVL in magenta, the host filamentous actin with phalloidin in grey, Hoechst is in cyan. **(D)** IF in intestinal tissue section of mice infected with SG4-HA. PanCp in magenta labels the parasite, phalloidin in grey the host F-actin and Hoechst the host and parasite nuclei. The HA in green showed a ring structure at the interface similar to the localization seen *in vitro*. **(E)** IF showing a sporozoite, labelled in magenta with Cp23, invading HCT-8 cells. Phalloidin is used to confirm the invasion by detecting the host actin polymerization dot. The HA staining in green is labelling SG4 being secreted at the infection site. **(F)** U-ExM on intestinal section using BODIPY in magenta to label the lipids (HPM, PPM, PV, FO) as well as NHS-ester in grey to label structure enriched in free amine like the dense band. SG4 labels the interface as a ring above the dense band in trophozoite as seen on projection (top) and single Z- slice (bottom). **(G)** U-ExM on intestinal section using HPA in yellow as a vacuole marker as well as NHS-ester in grey to label structure enriched in free amine like the dense band. SG4 labels the interface as a ring above the dense band in trophozoite at the base of the parasitophorous vacuole. **(H)** U-ExM on intestinal section using BODIPY in magenta to label the lipids (HPM, PPM, PV, FO) as well as NHS-ester in grey to label structure enriched in free amine like the dense band and the dense granules in meronts. SG4 is visible in the small granules as well as at the interface.

To validate the pull-down results, two of the strongest DG8-interacting candidates were selected for tagging. Attempts to tag the putative dense granule protein cgd4_3530 were unsuccessful as no parasites were recovered (S2B Fig). In contrast, tagging of cgd6_3920 was successful and U-ExM revealed its localization to the small granules in sporozoites (Figs 3A, 3B, S2C, and S2D) and was therefore named SG4 [16,23]. In HCT-8 cells and in mouse intestine, SG4 displayed a similar localization as DG8, forming a ring structure that appeared slightly thicker and extended toward the apical side of the vacuole (Fig 3C and 3D). As expected, SG4 relocalized to the apical region prior to invasion, was secreted during invasion and later accumulated in newly formed small granules within meronts (Figs 3C, 3E, and S2E). U-ExM on tissue confirmed SG4 localization above the DB, excluded from the FO, with extensions along the PVM (Figs 3F-3G and S2F-S2H). Interestingly, tissue sections also enabled visualization of post-fertilization stages, where an intracellular oocyst was observed with SG4 accumulating in the small granules of the four sporozoites and at the interface (S2I Fig). Next, SG4 function was assessed by genetic disruption. Direct knockout via insertion of a nanoluciferase and neomycin cassette into the coding sequence failed, as no parasites were recovered from mice, whereas parasites with an HA tag were viable (S2J Fig). The recently optimized DiCre-LoxP system was then applied [27]. Because SG4 lacks introns suitable for LoxP insertion, we attempted to introduce an artificial intron at two different positions (+1,614 or +1,300 nt). In both cases, no parasites were recovered, suggesting that either the artificial intron or the recodonization itself impaired survival by mimicking a gene knockout (S2K Fig). Similarly, attempts to append a miniTurboID tag at the C-terminus failed to yield parasites, likely due to disruption of secretion and protein function (S2L Fig). Together, these findings identify SG4, a putative DG8 partner, as a small granule protein that accumulates in a ring structure at the host-parasite interface and is likely essential for parasite survival *in vivo*.

### Antibodies against SG4 and DG8 label the interface

**Fig 4:**
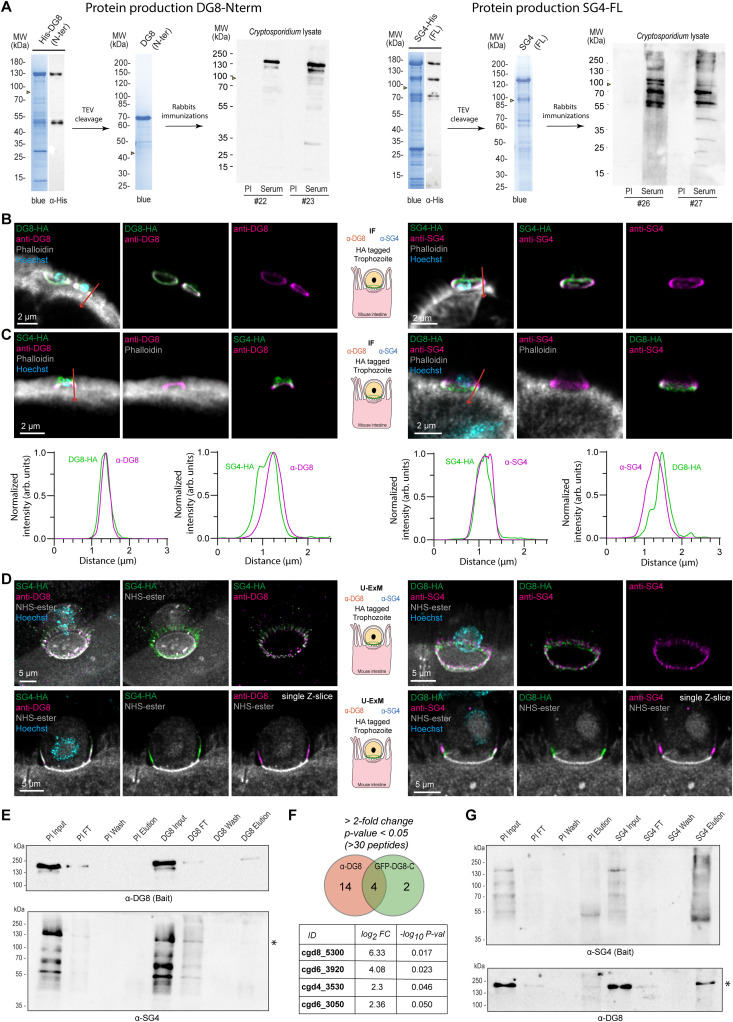
Antibodies against DG8 and SG4 reveal a partial colocalization at the interface. **(A)** Coomassie blue and anti-His western blot of the recombinant protein DG8-Nterm (left) and SG4-FL (right) without signal peptide showing a migration higher than the expected size indicated by the arrow heads even after removal of N-terminal tag by proteolytic cleavage using Tobacco Etch Virus (TEV) protease. Two pre-immune (PI) and sera from immunized rabbits were blotted on *Cryptosporidium* sporozoite lysate. Nothing was detected in the PI while the sera showed bands higher than the predicted size (90 and 91kDa shown by arrow heads). **(B)** IF showing a colocalization between the sera in magenta (#22 or #26) and the corresponding HA version in green (DG8 or SG4) from intestinal section. Hoechst in cyan and phalloidin in grey. **(C)** Top, IF on intestinal section using the sera anti DG8 (magenta) on SG4-HA (green) intestine (left) or using the sera anti SG4 (magenta) on DG8-HA (green) infected tissue (right). In both cases, SG4 labelling is partially colocalizing with DG8 and showing apical extension. Bottom, measurements of normalized intensity of DG8 and SG4 in (B) and (C) along the red arrow. **(D)** U-ExM on tissue showing on Z-projection (top) or single Z-slice (bottom) the partial colocalization of the sera in magenta (DG8 on the left or SG4 on the right) with the HA staining of SG4 on the left and DG8 on the right (green). **(E)** Pull down experiments from anti-DG8 antibodies or the pre-immune control showing the enrichment of DG8 (top) and SG4 (bottom) in the DG8-elution fraction. Asterix highlights the eluted SG4 protein. **(F)** Top, comparison of the proteins enriched in the anti-DG8 pull down (2-fold enrichment, p value < 0.05 and more than 30 peptides) with the GFP-DG8-C presented in Fig 2G. Bottom, list of the 4 common proteins with the corresponding enrichment and p-value from the anti-DG8 experiment compared to the pre-immune control. **(G)** Reverse pull down experiments from anti-SG4 antibodies or the pre-immune control showing the enrichment of SG4 (top) and DG8 (bottom) in the SG4-elution fraction. Asterix highlights the eluted DG8 protein.

To develop tools for precise localization of DG8 and SG4, polyclonal antibodies against both proteins were generated. A full-length recombinant protein of SG4 (excluding the signal peptide, N24–K789; MW: 89 kDa) and a truncated N-terminal fragment of DG8 (excluding the signal peptide, S25–G403; MW: 42.3 kDa) were produced, as the full-length DG8 could not be expressed. Both recombinant proteins migrated higher than the predicted sizes on acrylamide gels (>30 kDa shift) for reasons that remain unclear, but their identities were confirmed by mass spectrometry and their integrities by western blot with anti-His antibodies (Figs 4A and S3A). Rabbits were immunized with untagged recombinant proteins, and the resulting sera were tested against *Cryptosporidium* lysates (Fig 4A). Anti-DG8 sera (#22 and #23) detected two bands between 100–130 kDa, compared to the predicted 90 kDa size of endogenous DG8. Anti-SG4 sera (#26 and #27) labeled bands around 100 kDa, and higher as well as lower bands were observed, consistent with degradation products also seen in the recombinant SG4 purification (Fig 4A). At the same dilution and exposure time, the pre-immune sera failed to detect any *Cryptosporidium* sporozoite proteins. All immune sera reacted against wild-type parasites by microscopy, showing accumulation in the dense or small granules as well as at the interface. Sera with the lowest background were selected for subsequent experiments (sera #22 for DG8, sera #26 for SG4) (S4A and S4B Fig). To confirm specificity, the sera were tested by IF and U-ExM on parasites expressing the corresponding HA-tagged proteins. Clear colocalization between serum staining and anti-HA was observed in sporozoites by U-ExM (S4C Fig), in intestinal sections by IF (Figs 4B and S5A) and by U-ExM (S4D Fig). Furthermore, anti-DG8 serum showed no staining in the DG8-KO strain compared with the HA-tagged control, validating both the antibody and the mutant line (S4E Fig).

### *Cryptosporidium* secreted proteins form an organized layered interface

With specific antibodies in hand, the spatial relationship between DG8 and SG4 was examined. Using anti-DG8 antibodies on intestinal sections infected with SG4-HA parasites, a partial colocalization was observed, with SG4 staining extending above the DG8 ring (Figs 4C and S5B left). Reciprocal staining with anti-SG4 on DG8-HA sections confirmed this organization (Figs 4C and S5B right). Measurements from top to bottom of the infection site confirmed these observations (Figs 4B, 4C, S5A, and S5B, arrows). U-ExM further resolved these differences, revealing overlapping but distinct distributions, with SG4 showing longer apical extensions relative to DG8, clearly visible in single Z-section (Figs 4D and S5C). To confirm the interaction of these proteins at the interface of WT parasites with HCT-8 cells, a pull down with anti-DG8 antibodies was performed. Endogenous DG8 proteins were pulled down with the anti-DG8 sera (Fig 4E) along with a faint detection of SG4 by western blot that was confirmed by mass spectrometry. A total of 18 proteins were enriched in the DG8 conditions (>2-fold change, p-value<0.05 and >30 PSMs) including the top four proteins of the pull-down from GFP-DG8-C ectopic expression (Fig 4F). The additional 14 putative partners were enriched in proteins with localization prediction to the dense and small granules (8 out of 14) suggesting a larger complex (S1 Table). The interaction of SG4 with DG8 was then confirmed by performing a reverse pull-down from the anti-SG4 sera and pulled down DG8 as shown by western blot (Fig 4G).

**Fig 5:**
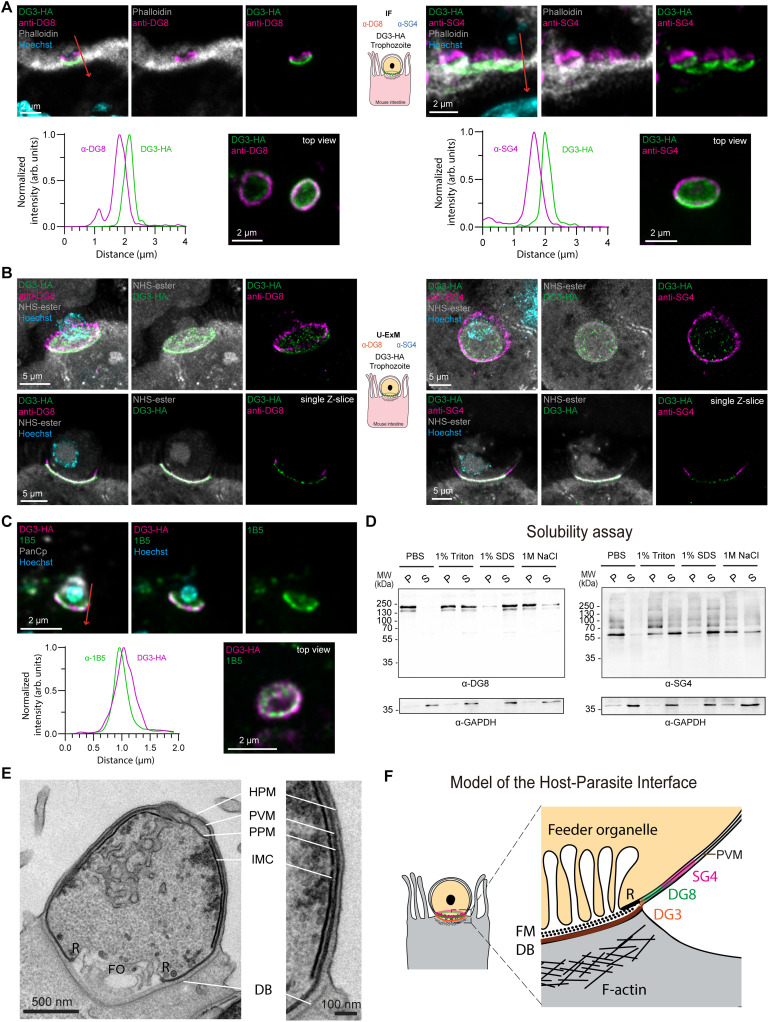
DG8 and SG4 form a complex at the parasitophorous vacuole membrane while DG3 accumulates in the dense band. **(A)** Top, IF on intestinal section showing in magenta sera anti-DG8 (left) or anti-SG4 (right) higher than the HA staining (in green) labelling DG3-HA. Phalloidin in grey labels the intestinal brush border, Hoechst in cyan labels nuclei. Bottom, measurements of normalized intensity of DG3 and DG8 or SG4 along the red arrow and an additional image showing an infection from the top view. **(B)** U-ExM using the sera anti-DG8 (left) or the sera anti-SG4 (right) in magenta of intestinal section of DG3-HA (green) on a Z-projection (top) or single Z-section (bottom). DG3 accumulates at the NHS-ester positive structure (grey) corresponding to the dense band below the SG4 and DG8 staining. Hoechst in cyan labels nuclei. **(C)** Top, IF on intestinal section labelling the parasite with PanCp in grey showing a colocalization of DG3-HA in magenta and 1B5 in green; Hoechst in cyan labels nuclei. Bottom, measurements of normalized intensity of DG3 and 1B5 along the red arrow and an additional image showing an infection from the top view. **(D)** Solubility assay with PBS, 1% Triton X-100, 1% SDS and 1 M NaCl on WT parasites infecting HCT-8 cells for 22 hours and revealed with anti-DG8 (left) or anti-SG4 (right). GAPDH was used on the same blot as control of solubilization of the samples. **(E)** Electron Microscopy image of *Cryptosporidium* infecting HCT-8 for 5 hours highlighting the tight space between the HPM, the PPM and the PVM. **(F)** Model of the localization of SG4, DG8 and DG3 relative to known structures at the interface adapted from Guérin and Striepen, 2020. FM, Filamentous mesh; DB, Electron-dense band; R, Electron-dense ring-shape structure; FO, Feeder organelle; HPM, Host plasma membrane; PVM, Parasitophorous vacuole membrane; PPM, Parasite plasma membrane; IMC, Inner membrane complex.

The localization of DG8 and SG4 were then compared with DG3, another secreted protein known to accumulate at the host-parasite interface [16] that was also enriched in the anti-DG8 pull down experiment (2-fold change = 2.35; p-value = 0.028; PSMs = 58) (S1 Table). We confirmed, as previously reported [16], that DG3 was detected as a cup-like structure rather than a ring at the host-parasite interface both *in vitro* and *in vivo* (S6A and S6B Fig). In DG3-HA infected mice, both DG8 and SG4 antibodies labelled rings positioned above the DG3 cup-like structure suggesting a layered structure (Figs 5A and S6C). U-ExM further localized DG3 to the NHS-ester stained structure at the interface, which we believe corresponds to the electron-dense band (Figs 5B and S6D). In the search to identify markers for the host-parasite interface, we reinvestigated the monoclonal antibody 1B5 of unknown target, previously reported to label the host-parasite interface [28]. Based on its mid-sporozoite staining pattern, we hypothesized that 1B5 recognizes an unknown dense granule protein. Indeed, U-ExM on wild-type sporozoites showed that 1B5 accumulates in dense granules labeled by NHS-ester and colocalizes with DG8 in DG8-HA parasites (S7A Fig). To examine its localization in tissue, we applied 1B5 to intestinal sections, including a blocking step with goat anti-mouse antibody in 10% BSA before adding the 1B5 antibody to block the existing mouse antibody in the tissue section. This revealed a partial colocalization of 1B5 with DG3, positioned below DG8 (Figs 5C, S7B and S7C). Finally, the use of 1B5 and anti-SG4 antibodies demonstrated that, in the DG8-KO parasites, both SG4 and the protein recognized by 1B5 maintain their localization at the interface suggesting that these structures are not disrupted in the absence of DG8 (S7E and S7F Fig).

### DG8 and SG4 are associated with membranes

Because western blotting of transgenic parasites requires large parasite numbers, the antibodies were used on wild-type parasites to examine the solubility of endogenous DG8 and SG4. This approach allowed to assess their potential association with membranes (1% Triton X-100) or with other proteins via ionic interactions (1M NaCl). HCT-8 cells were infected with wild-type parasites for 22 hours, harvested and incubated for 1 hour in four different buffers after flash freezing to lyse the infected cells. Supernatants and pellets were separated and analyzed by SDS-PAGE (Fig 5D). DG8 and SG4 displayed similar solubility profiles. Neither protein was soluble in PBS and minimally in high salt (1M NaCl) while they were solubilized by strong denaturing conditions (1% SDS). Both proteins became partially soluble upon addition of 1% Triton X-100, consistent with membrane association. Together with the microscopy images, we suspect both proteins to be localized to the PVM. Electron microscopy of intracellular trophozoites further illustrates the challenge of assigning proteins to specific sub compartments, as the host, parasite and parasitophorous vacuole membranes are tightly apposed (Figs 5E and S7G). Taken together, U-ExM on tissue, combined with *Cryptosporidium*-specific antibodies for dense (1B5, DG8) and small granules (SG4), along with complementary biochemical approaches, provided a refined characterization of the multilayered host-parasite interface (Fig 5F).

## Discussion

Apicomplexans have evolved diverse mechanisms to modify and exploit their host cells. Among them, *Cryptosporidium* stands out due to its unusual epicellular localization, yet much remains unknown about it. Our work aimed to characterize the host-parasite interface both at the molecular level and through imaging. Ultrastructure expansion microscopy has transformed parasitology by enabling unprecedented resolution with a technique accessible to everyday laboratories [29] but the lack of specific antibodies for *Cryptosporidium* had long been a limitation. The combination of dyes with U-ExM recently overcame this barrier, allowing localization of tagged proteins within distinct secretory organelles [16] in a reproducible manner [17,19,20,23,30,31]. Building on this, we applied U-ExM on intestinal sections from infected mice, revealing structures previously only accessible by electron microscopy. In addition to labeling secretory organelles, NHS-ester staining highlights the electron-dense band, an enigmatic structure at the interface. This band has previously been detected with anti-*Cryptosporidium* serum, indicating its parasite protein-containing composition [32], though specific constituents had not been described. Here, we assign the previously studied dense granule protein DG3 and the DG protein recognized by monoclonal antibody 1B5 as components of this structure. Additional proteins, likely of both parasite and host origin, remain to be identified. Furthermore, defining the target of 1B5 alongside reassessing known effectors at the interface will be essential for our understanding of this structure.

Our findings confirm that secreted effector protein destination sites are not strictly determined by the organelle of origin. Proteins originating from dense granules, small granules and likely rhoptries form complexes at multiple locations. With DG8 and SG4 antibodies, we can now label both their respective organelle and their position at the interface. The lack of overlap between these markers in the sporozoites confirms that small granules are not precursors of dense granules [16], however the recent identification of MUC5 in the small and dense granules [31] suggests proteins shared by both organelles exist. While both dense and small granules are secreted during invasion, small granules appear to be constitutively secreted, potentially maintaining structures such as the electron-dense band and the electron-dense ring-shape structure, whereas dense granules may drive their initial formation. However, it has been shown that small granules can have roles independent of dense granules as shown recently in the modification of the host microvilli [30,31]. Interestingly, DG8 seems to be only present in *Cryptosporidium* species infecting the intestine and not those infecting the stomach. These open questions about the adaptation of *Cryptosporidium* to its niche with parasite specific effectors.

A central question in the field remains the number and origin of membranes at the parasite-host interface [8]. The feeder organelle (FO) forms a single membrane barrier between the parasite and host cytoplasm, but whether it derives from the parasite plasma membrane, the parasitophorous vacuole membrane, or a fusion of both remains unresolved. Identification of protein markers for each membrane compartment would provide valuable insight into the interface dynamics during *Cryptosporidium* infection. Another open question is whether the dense band contains membrane or is purely proteinaceous. Using BODIPY to label membranes, we detected no lipid bilayer at the dense band, though this approach potentially lacks resolution. Together with EM observations, our data support the idea that the dense band is devoid of lipids and that the FO represents the sole membrane separation between host and parasite cytoplasm, functioning as a hub for transporters and nutrient acquisition [20,25]. To add further complexity, the interface is dynamic, remodeling throughout the 12 hours of intracellular replication [25,33]. Additional structures observed by EM, including a filamentous mesh and the electron-dense ring-shape structure where parasite plasma membrane and parasitophorous vacuole membrane merge into the FO, will also require further characterization.

Here, we showed that two secretory proteins, DG8 from the dense granules and SG4 from the small granules, form a ring structure at the host-parasite interface. While we couldn’t deplete SG4 with our current depletion system, DG8 was successfully knocked out by delivering two guides simultaneously to remove the entire coding sequence. The depletion of DG8 did not affect the localization of SG4, nor the overall structure of the host-parasite interface label by NHS-ester, but reduced infection *in vitro* and *in vivo*. The exact role of DG8 during infection establishment remains to be clarified and confirmed through complementation experiments but its function may be compensated for by other proteins that are part of the same complex. Microscopy, biochemical analyses and antibody staining suggest that DG8 and SG4 might be part of a larger complex with additional secreted proteins including the putative dense granules cgd4_3530 and cgd6_3050. Although U-ExM improves resolution, the precise localization of membrane within or in proximity of this complex remains unclear. Neither DG8 nor SG4 are predicted to possess a transmembrane domain, however they require detergent to become soluble, suggesting that they are embedded into membranes. One possibility would be that association is mediated via GPI anchors or by interacting with membrane-associated proteins, although the latter hypothesis is less likely because proteins are not fully solubilized by high NaCl concentrations. Future work will be required to confirm whether the additional putative DG8 interactors identified in our pull-downs are part of the same complex.

The localization of DG8 was assessed during the revision of our manuscript by an independent group [23] and detected as a ring at the base of the PVM by IF and immuno-gold labelling confirming our observations. The immuno-gold labelling, similar to our U-ExM, failed to accurately resolve in which membrane, PVM or HPM, the protein accumulates while it’s most likely the PVM. It will be important to develop and adapt tools to understand and separate these two membranes as it will not only have an impact on the immune system exposure, but also on the secretion and trafficking to the different membranes. Interestingly, DG8 as well as SG3 and DG7 have been detected upregulated when CP2, a small granule protein accumulating at the PVM, was knocked out [23]. In addition, the authors showed a layered accumulation of DG8, CP2 and SG3 along the PVM. We detected CP2 (cgd6_5410) and SG3 (cgd6_5400) enriched in our DG8 pull down experiment (2-fold change = 3.62 and 2.84; p-value = 0.017 and 0.026 respectively) (S1 Table) suggesting that they might transiently associate during the PVM formation. It would be meaningful to assess if the transcript of CP2 is upregulated in the absence of DG8. Interestingly, the pull down was also enriched, at a relatively low peptide count, with DG4, DG5, ROP3 and ROP7 previously detected in the PV [16,18,19].

In conclusion, our work introduces powerful new tools and provides insights into the molecular architecture of the *Cryptosporidium*-host interface. Importantly, the interface, and especially the DG8/SG4-associated ring, seems critical for parasite survival. Deletion of DG8 strongly reduces parasite fitness, while SG4 is likely essential, making this structure a promising target for intervention. Altogether, these findings advance our understanding of how the parasite exploits its host and highlight structures that appear essential for the parasite’s survival.

## Materials and methods

### Ethics statement

All animal studies were conducted in compliance with the Swiss National Institutional Guidelines on Animal Experimentation and were approved by the Swiss Cantonal Veterinary Office Committees of Geneva for Animal Experimentation (GE276D).

### Parasite strains

*C. parvum* oocysts (Iowa II strain) used in this study were purchased from the Bunchgrass Farms Deary, ID.

### Host cell culture

HCT-8 (ATCC: CCL-244) and HEK293T-17 (ATCC: CRL-11268) cells were maintained in Dulbecco’s Modified Eagle Medium (DMEM) supplemented with 10% bovine serum at 37°C with 5% CO2. HEK293T-17 cells were used for transient transfection, while HCT-8 cells served as host cells for *C. parvum* infections.

### Mouse model

Ifng^-/-^ mice on a C57BL/6J background were purchased from Charles River Laboratories and bred at the University of Geneva. Mice aged 6–15 weeks were used for infections with various *C. parvum* transgenic lines.

### Generation of transgenic parasites

*C. parvum* oocysts were excysted and electroporated as previously described [16,34]. Briefly, a guide RNA targeting the 3’UTR of the gene of interest was inserted by ligation in the Cas9 vector. For transfection of HA tagged strains, 50 µg of this plasmid was co-precipitated with 200 µL of repair template PCR containing 30 bp homology arms to the respective gene, a triple hemagglutinin (HA) tag, a generic 3’ UTR and a nanoluciferase-neomycin cassette driven by the enolase promoter **(primers listed in** S2 Table). To generate the KO line, the original Cas9 plasmid was modified to express two gRNAs targeting the beginning of the gene and 3’UTR simultaneously, while using a similar repair template as above. Excysted sporozoites were transfected with the Cas9-gRNA vector and repair template. To artificially induce excystation, 20 million *C. parvum* oocysts were treated with hydrochloric acid, then washed and resuspended in 0.2 mM sodium taurodeoxycholate and 20 mM sodium bicarbonate. Transfected parasites were gavaged into Ifng^-/-^ mice, and resistant transformants were selected by supplementing drinking water with paromomycin. Oocysts shed in feces were collected and purified by sucrose floatation and cesium chloride gradient, then stored in PBS at 4°C until further use.

### Integration PCR

Parasite genomic DNA (gDNA) were extracted using the Quick-DNA Fecal/Soil Microbe Microprep kit. For HA-tagged strains, PCR primers were designed outside the 5’ and 3’ homology arms as well as within the HA tag and the neomycin resistance cassette to confirm correct integration. For knockout (KO) strain, deletion of the protein-coding sequence was verified using primers within the targeted gene region, while correct integration of the repair template was assessed at both the 5’ and 3’ ends using primers binding to the enolase promoter and the neomycin resistance cassette. An independent strain was used as PCR control.

### Immunofluorescence Assay (IF) on cells and intestinal tissue sections

Bleach-treated oocysts were excysted with 0.8% sodium taurocholate for 10 min. To image sporozoites, excysted oocysts were incubated in DMEM for 1h at 37°C and then allowed to settle onto poly-L-lysine coated coverslips for 30 min. To investigate intracellular parasites, excysted sporozoites were used to infect HCT-8 cells for the time periods indicated in individual experiments. Cells were fixed with 4% paraformaldehyde for 15 min and permeabilized with 0.5% Triton X-100 for 15 min. Samples were blocked with 4% bovine serum albumin (BSA) for 1 hour at room temperature (RT), followed by incubation with primary antibodies diluted in 1% BSA for 1h at RT. After three washes with PBS, samples were incubated with secondary antibodies diluted in 1% BSA for 1 hour at RT **(antibodies and dyes dilutions are listed in** S3 Table). Nuclei were stained with Hoechst (diluted in PBS) for 5 min, followed by three PBS washes. Coverslips were mounted on glass slides with Fluoromount. For mouse intestinal tissue sections, samples were hydrated in 1% BSA/0.1% Triton X-100, blocked in 10% BSA/0.1% Triton X-100, and incubated with primary antibodies diluted in 1% BSA/0.1% Triton X-100 for 2h. After three washes in the same buffer, sections were incubated with secondary antibody for 1h 30 min, stained with Hoechst for 30 min, and mounted with Fluoromount. When the 1B5 antibody was used, goat antibodies against mouse not coupled to a fluorochrome were used in the blocking buffer to prevent the recognition of natural mouse antibodies present in the section. Imaging of both tissue section and cells was performed on Leica SP8 or Stellaris confocal microscopes. The plotting of the intensity profile was generated using ImageJ built-in function, numerical data were then exported in GraphPad to generate the graphical representations.

### Ultrastructure expansion microscopy (U-ExM) on cells and tissues sections

U-ExM was applied as described [35]. Parasites were excysted as described in the IF section. Samples were embedded overnight in an acrylamide/formaldehyde solution to anchor proteins. Gelation was induced with monomer solution supplemented with TEMED and APS for 1h at 37°C, followed by denaturation buffer at 95 °C. Gels were placed in water to expand around 4 times its size, and then shrunk in PBS before immunostaining. Samples were incubated with primary antibodies for 3h at 37°C, washed three times with PBS-Tween 0.5% and incubated with secondary antibodies containing Hoechst for 2h 30 min at 37°C in PBS before final expansion in water. When used, NHS-ester was incubated in PBS for 2h 30 min while BODIPY was diluted in water. For tissue sections, OCT was removed from sections by PBS washes. Sections were incubated in the same acrylamide/formaldehyde anchoring solution for 3h at 37°C before incubating the section with monomer solution without APS or TEMED, allowing the penetration of the solution within the tissue for 1h 30 min at RT. 45 µL of monomer solution containing the APS and TEMED were added on the section and a coverslip was used to flatten the solution for 45 minutes on ice before overnight gelation at 37°C. Gels were denatured for 90 min at 95°C, expanded in water and processed for immunostaining as described above. Imaging of expanded samples was performed with Leica SP8 confocal microscope.

### *In vitro* growth assay

HCT-8 cells in 96-well plates were infected with 10,000 oocysts per well of DG8-HA or DG8-KO transgenic strains. At 24h and 48h post-infection, cells were washed with PBS, lysed with NanoLuc lysis buffer supplemented with 1 M DTT, and luminescence was measured using the Nano-Glo Luciferase Assay (Promega) on a Glomax plate reader. Three independent biological replicates were performed, each in technical triplicates.

### Virulence assay *in vivo*

Groups of three litter mice (6–8 weeks old) were infected with 10,000 oocysts of either DG8-HA or DG8-KO strains and monitored for 18 days. Fecal samples were collected every other day and parasite burden was quantified by measuring luminescence in 20 mg of feces. The assay was performed in triplicate.

### 24 hours quantification assay

HCT-8 cells seeded in 24-well plates were infected with 100,000 oocysts of DG8-HA or DG8-KO in parallel. At 2h post-infection, cells were fixed and immunostained as described in the IF section. Parasite numbers were quantified from 40 fields of view acquired using a widefield Axiocam Fluo microscope. Three technical replicates were performed.

### Solubility assay

Solubility Assay was performed using PBS, 1% Triton X-100, 1 M NaCl, or 1% SDS. HCT-8 cells seeded in 24-well plates were infected with 2 million *C. parvum* oocysts per condition for 22h. Cells were washed with cold PBS and resuspended each in 100 µL of the indicated buffer, flashed freeze in liquid nitrogen at least 5 times and incubated on ice for 1 hour. Samples were centrifuged and the supernatant was separated from the pellet. Pellets and supernatants were resuspended in Laemmli buffer containing 0.1 M DTT to a similar volume. Samples were boiled for 10 min at 95° C, resolved by SDS-PAGE and analyzed by western blotting.

### Protein and antibody production

The full-length sequence of SG4 (N24-K789) and DG8 (S25-N816) recodonized cloned into bacterial expression plasmids were ordered from Twist for expressed in *E. Coli* Lobst-R cells. The full-length sequence of SG4 (N24-K789) recodonized for human codons was fused to a C-terminal Twin-STREP-His tag preceded by a TEV protease recognition site. The N-terminal region of DG8 (S25-G403) was fused to a N-terminal His-MBP tag containing a TEV protease recognition preceding the protein coding sequence. Both proteins were produced following the same procedure. Proteins were expressed in *E.coli* following standard protocols. The harvested cells were lysed in lysis buffer (50 mM Tris pH 7.5, 1.000 mM NaCl, 10 mM Imidazole, 3 mM beta-mercaptoethanol) using LM-20 microfluidizer and the lysate was centrifuged at 35.000 g for 35 min at 4 °C to remove cell debris and membranes. The soluble fraction was applied to a 5 mL His-trap column. The column was washed with lysis buffer, and the proteins were eluted with elution buffer (50 mM Tris pH 7.5, 500 mM NaCl, 350 mM Imidazole, 3 mM beta-mercaptoethanol). 1 mg of His-TEV protease was added to the eluted proteins, and the sample was dialyzed against 1 L of dialysis buffer (PBS supplemented with 300 mM NaCl and 3 mM beta-mercaptoethanol). Cleaved proteins were applied to a 5 mL His-trap column to capture uncleaved proteins, the tag and the protease while the untagged proteins were collected from the flow-through. Purified non-tagged proteins were shipped to Eurogentec for rabbit immunization. Pre-immune sera were first tested for cross-reactivity against *Cryptosporidium*, and two suitable rabbits per antigen were selected for immunization. Immunization was carried out in five rounds. The medium bleed was collected on day 66 after four injections, and the final bleed was collected on day 122 following a fifth immunization on day 84.

### Transient transfection of HEK293T-17

The nucleotide sequence of DG8 was recodonized, human-optimized and synthesized by Twist Bioscience. The Full-length, N-terminal or C-terminal region of DG8 was cloned into a CMV-GFP vector using Gibson Assembly. For GFP Trap assays, HEK293T-17 cells were transiently transfected with the recodonized DG8-C terminus fused to an N-terminal GFP tag or with GFP alone as a negative control, using lipofectamine 3,000 (Thermo Fisher Scientific). Opti-MEM was used as transfection medium and was changed to standard DMEM 4h post transfection. After 24h incubation, transfection efficiency was assessed and GFP pull down assay was performed (see section GFP Trap).

### GFP trap

One T25 flask of HCT-8 cells was infected with 80 million *C. parvum* oocysts for 3h, washed with PBS and combined with GFP-DG8-C or GFP transfected HEK293T-17 cells. For each condition (Negative control GFP or GFP-DG8-C), half a T25 flask of infected HCT-8 cells and two P6 wells of transfected HEK293T-17 cells were used. Cells were lysed in 250 µL Pierce IP buffer (Thermo Fisher Scientific) supplemented with protease inhibitor, followed by sonication and incubation on ice for 30 min. The supernatant was diluted with 250 µL cold wash buffer (50 mM Tris-HCl pH 7.5, 150 mM NaCl, 0.02% Tween-20, 0.5 mM EDTA, protease inhibitor) and incubated with 60 µL of GFP Trap magnetic agarose beads (Chromotek) for 3h at 4°C under rotation. The beads were washed beforehand with the same wash buffer. Following incubation, beads were washed three times with wash buffer and finally three times with PBS to remove detergent. The efficiency of pull down was checked by western blot (see section Western blot analysis). The GFP Trap pull down was performed in technical triplicates. Protein identification on the immunoprecipitated samples were performed by mass spectrometry-based analysis (see section LC-ESI-MS/MS analysis).

### Pull down from Sera

Pull down assays with DG8 and SG4 sera were performed using Dynabeads Protein G (Invitrogen). Pre-immune serum served as negative control. For each condition, two wells of HCT-8 cells in a 6-well plate were infected with 150 million *C. parvum* oocysts. At 3h post-infection, cells were washed with PBS and lysed in 250 µL Pierce IP buffer (Thermo Fisher Scientific) supplemented with protease inhibitor, followed by sonication and incubation on ice for 30 min. The supernatant was diluted with 250 µL cold wash buffer (50 mM Tris-HCl pH 7.5, 150 mM NaCl, 0.02% Tween20, 0.5 mM EDTA, protease inhibitor). 50 µL of Protein G magnetic beads were washed three times with PBS + 0.02% Tween-20, incubated with 10 µL of DG8/SG4 serum or pre-immune serum for 3h at 4°C and subsequently washed three times with the wash buffer to remove unbound antibodies before being incubated with the lysate for 3h at 4°C under rotation. Beads were then washed three times with wash buffer and three times with PBS to remove detergent. Pull-down efficiency was assessed by western blotting. Immunoprecipitated proteins from DG8 sera were further analyzed by LC-ESI-MS/MS in technical triplicates.

### Western blot analysis

For western blotting, samples were resuspended in 2x Laemmli containing SDS and boiled at 95°C for 10 min. Proteins were separated on a 12.5% polyacrylamide gel by SDS-PAGE and transferred to a nitrocellulose membrane (0.45 µm pore size; Cytiva). Membranes were blocked with 5% milk in PBS-0.05% Tween-20 and incubated with primary antibody diluted in blocking buffer for 1h. After three washes in PBS-0.05% Tween-20, membranes were incubated with HRP-conjugated secondary antibody for 1h. Protein bands were detected using chemiluminescence.

### LC-ESI-MS/MS analysis

On-bead digestion of proteins was performed using Trypsin/LysC with the iST kit (PreOmics). Resulting peptides were analyzed by nano LC-ESI-MS/MS on an Easy-nLC 1,000 liquid chromatography system (Thermo Fisher Scientific) coupled to a Q-Exactive HF Hybrid Quadrupole-Orbitrap mass spectrometer. Database research was carried out with Mascot (Matrix Science) against *Cryptosporidium parvum* reference proteome (Uniprot ID UP000006726), including bait protein sequences. Data was processed and validated using Scaffold (Proteome Software) with a protein false discovery rate (FDR) cutoff of 1% and a minimum of two unique peptides per protein. The mass spectrometry proteomics data have been deposited to the ProteomeXchange Consortium via the PRIDE partner repository with the dataset identifier PXD074689 and 10.6019/PXD074689. The results are summarized in S1 Table.

### Electron Microscopy (EM)

For EM analysis, HCT-8 cells seeded on 12 mm diameter round glass coverslips in 24-well plate were infected with 10 million *C. parvum* oocysts per well for 5h. At 3h post-inoculation, extracellular parasites were removed by washing with PBS. Cells were fixed with 2.5% glutaraldehyde and 2% paraformaldehyde for 1h at room temperature, washed once in PBS and kept in PBS. Fixed samples were extensively washed with 0.1 M sodium cacodylate buffer and prepared as previously described [36]. Laser microdissection microscope (Leica Microsystems) was used to select suitable areas and to outline their positions on the resin surface. 70 nm ultrathin serial sections were cut with a diamond knife (DiATOME) and sections were examined by Tecnai 12 G2TEM (FEI) operating at 80 kV of acceleration voltage and equipped with a side-mounted MegaView III CCD camera (Olympus Soft-Imaging Systems) controlled by iTEM acquisition software (Olympus Soft-Imaging Systems) at the Electron Microscopy Facility (PFMU) at the Faculty of Medicine at the University of Geneva.

### Statistical analysis

Statistical tests were done on GraphPad Prism. Unpaired t-test was used as shown in figure legends and raw data are provided in S4 Table.

## Supporting information

S1 Fig DG8 additional characterization. **(A)** Single cell from Walzer et al., 2024 and hyperLOPIT information from Guérin et al., 2023 for the candidate cgd8_5300 supporting its localization in the dense granules. **(B)** Left, schematization of the tagging strategy for cgd8_5300 (DG8) to introduce a triple HA tag followed by a nanoluciferase neomycin cassette. Right, gel of the integration PCR. **(C)** Additional U-ExM image of excysted DG8-HA sporozoite on poly-L-lysine (corresponding to Fig 1B right). **(D)** Additional U-ExM images of DG8-HA trophozoites in intestinal section using BOPIDY and NHS-ester (corresponding to Fig 1G). **(E)** Additional U-ExM image of DG8-HA trophozoite in intestinal section using HPA and NHS-ester (corresponding to Fig 1H). **(F)** Additional U-ExM image of DG8-HA meront in intestinal section using BODIPY and NHS-ester (corresponding to Fig 1I). **(G)** Left, schematization of KO strategy for DG8 with guide 1 targeting the 5’UTR of the gene and a guide 2 targeting the 3’UTR of the gene. Right, integration PCR of the WT versus KO of DG8 showing a complete absence of WT version in the population. **(H-I)** The two additional experiments for virulence assays (Area under the curve = 11-fold decrease on upper graph and 13.5-fold on lower graph) and growth assays (fold change at 24h = 1.9, at 48h = 3.5 on upper graph and fold change at 24h = 6.6, at 48h = 7.3 on lower graph) corresponding to Fig 2B-2C. **(J)** Schematization and correct PCR integration of the miniTurboID strategy for DG8. **(K)** IF on DG8-HA versus DG8-turboID-HA shows an absence of HA detection in the miniTurboID strain suggesting that the addition of a large tag affected the secretion of DG8 and potentially its expression. VVL in magenta labels the parasite vacuole, Hoechst the nucleus and HA the protein DG8 in green. **(L)** U-ExM of invading DG8-HA (left) or DG8-KO (right) in HCT-8 cells using PanCp to label the parasites and NHS-ester the interface. Hoechst in cyan labels nuclei.
(TIF)

S2 Fig SG4 additional characterization. **(A)** Stage specific transcript from Tandel et al., 2019, single cell from Walzer et al., 2024 and hyperLOPIT information from Guérin et al., 2023 of the candidates cgd6_3920 (top) and cgd6_5270 (bottom) suggesting a small granule and rhoptry protein respectively. **(B)** Left, schematization of the tagging strategy for cgd4_3530 to introduce a triple HA tag followed by a nanoluciferase neomycin cassette. Right, luciferase from 20 mg of feces shows an absence of parasite growth. **(C)** Left, schematization of the tagging strategy for cgd6_3920 (SG4) to introduce a triple HA tag followed by a nanoluciferase neomycin cassette with the gel of the integration PCR. Right, luciferase from 20 mg of feces showing the growth of the parasite. **(D)** Additional U-ExM image of excysted SG4-HA sporozoite on poly-L-lysine (corresponding to Fig 3B). **(E)** IF showing a sporozoite, labelled in magenta with Cp23, invading HCT-8 cells. The HA staining in green labels SG4 being relocated apically before secretion while the actin patch is not formed yet (phalloidin in grey). **(F)** Additional U-ExM images of SG4-HA trophozoites in intestinal section using BOPIDY and NHS-ester (corresponding to Fig 3F). **(G)** Additional U-ExM image of SG4-HA trophozoite in intestinal section using HPA and NHS-ester (corresponding to Fig 3G). **(H)** Additional U-ExM image of DG8-HA meront in HCT-8 using BODIPY and NHS-ester (corresponding to Fig 3H). **(I)** U-ExM on intestinal section using NHS-ester in grey to label structures enriched in free amine like the dense band and the dense granules in sporozoites as well as a structure resembling the oocyst wall suture [37]. SG4 is visible in the small granules as well as at the interface. **(J)** Schematization of the KO strategy for SG4 that was transfected into sporozoite with the luciferase from 20 mg of feces showing an absence of parasite growth. **(K)** Schematization of the inducible KO strategy for SG4 with the luciferase from 20 mg of feces shows an absence of parasite growth. **(L)** Schematization of the tagging of SG4 with a miniTurboID with the luciferase from 20 mg of feces showing an absence of parasite growth.
(TIF)

S3 Fig Mass spectrometry of DG8 and SG4 recombinant proteins. Top, mass spectrometry on the recombinant protein DG8 shows peptides corresponding to the N-terminus of the protein in red. Bottom, mass spectrometry on the recombinant protein SG4 shows peptides corresponding to the full length of the protein without the signal peptide sequence in red.
(TIF)

S4 Fig Validation of DG8 and SG4 sera on WT and HA-tagged parasites. **(A)** U-ExM of excysted WT sporozoite showing the labelling of the sera anti-DG8 #22 (top left) and #23 (bottom left) in green with the dense granules (NHS-ester in grey) while the sera anti-SG4 #26 (top right) and #27 (bottom right) in green label the small granules perinuclear. When not mentioned otherwise, anti-DG8 corresponds to serum #22 and anti-SG4 to serum #26. **(B)** IF of 3 hours infected WT parasite in HCT-8 cells showing the detection of DG8 (left) and SG4 (right) using the generated sera as a ring structure in green. VVL in magenta labels the parasitophorous vacuole, phalloidin the F-actin and Hoechst the nuclei. **(C)** U-ExM of excysted sporozoite on poly-L-lysine show a colocalization between the HA version (green) and the sera version within the dense granules (anti-DG8 in magenta) on the left and within the small granules (anti-SG4 in magenta) on the right. NHS-ester in grey; Hoechst in cyan. **(D)** U-ExM on intestinal section using NHS-ester in grey to label the dense band, Hoechst in cyan and sera anti-DG8 or anti-SG4 in magenta. The sera colocalize with the HA staining in green. **(E)** Sera against DG8 (magenta) was tested on DG8-HA (left) and DG8-KO (right) using the same microscope settings. Intracellular parasite labelled in green (VVL, vacuole) with a host actin patch (phalloidin, grey) and their nuclei in cyan do not show a staining with the sera DG8 in the mutant strain while seen in the control strain confirming both the antibody specificity and the KO strain.
(TIF)

S5 Fig Analyses of DG8 and SG4 sera at the interface. **(A)** Additional IF images of the sera DG8 on intestinal section of DG8-HA (left) and sera SG4 on intestinal section of SG4-HA (right) (corresponding to Fig 4B). **(B)** Top, additional IF images of the sera DG8 on intestinal section of SG4-HA (left) and sera SG4 on intestinal section of DG8-HA (right) (corresponding to Fig 4C). Bottom, plot of normalized intensity of DG8 and SG4 along the red arrow of image from A and B. **(C)** Additional U-ExM images of SG4-HA trophozoite in intestinal section using the sera DG8 (left) and DG8-HA trophozoite using the sera SG4 (right) (corresponding to Fig 4D).
(TIF)

S6 Fig DG3 at the interface. **(A)** Schematization and integration PCR for the tagging of DG3 (cgd1_590) as previously done in Guérin et al., 2023. **(B)** IF *in vitro* (top) and *in vivo* (bottom) showing the accumulation of DG3 (green) as a cup-like structure at the interface with the host cell. Magenta labels the parasite (VVL on top or PanCP on bottom) while phalloidin in grey labels the host F-actin and Hoechst in cyan the nuclei. **(C)** Top, additional IF images of the sera DG8 (left) or the sera SG4 (right) on intestinal section of DG3-HA (corresponding to Fig 5A). Bottom, plot of normalized intensity of DG8 or SG4 and DG3 along the red arrow of images from top. **(D)** Additional U-ExM images of DG3-HA trophozoite in intestinal section using the sera DG8 (left) or the sera SG4 (right) (corresponding to Fig 5B).
(TIF)

S7 Fig Monoclonal antibody 1B5 recognizes a dense granules protein at the interface. **(A)** Left, U-ExM of excysted WT sporozoite showing the labelling of the hybridoma 1B5 in the dense granules. Right, IF of 1B5 on DG8-HA strain showing that 1B5 localizes within DG8 labelled dense granules. **(B)** Top, additional IF image of 1B5 on intestinal section of DG3-HA (corresponding to Fig 5C). Bottom, plot of normalized intensity of 1B5 and DG3 along the red arrow of image from B. **(C)** Top, IF of 1B5 on intestinal section of DG8-HA showing that 1B5 (green) recognizes a protein more basal than the DG8 (magenta). Bottom, IF of 1B5 (green) and the anti-DG8 sera (grey) on intestinal section of DG3-HA (magenta) showing a colocalization of 1B5 and DG3 below DG8. **(D)** U-ExM of intestinal section of DG8-HA in magenta showing a colocalization of 1B5 in green with the NHS-ester positive dense band; Hoechst in cyan labels nuclei. **(E)** Sera against SG4 (top) and 1B5 (bottom) were used on DG8-HA (left) and DG8-KO (right) using the same microscope settings. Intracellular parasite labelled in green (VVL, vacuole) with a host actin patch (phalloidin, grey) and their nuclei in cyan (Hoechst) shows a similar staining for SG4 and 1B5 in both strains. **(F)** U-ExM of invading DG8-HA (top) or DG8-KO (bottom) in HCT-8 cells showing normal SG4 accumulation at the interface (green). Hoechst in cyan labels nuclei. **(G)** EM of 5h infected WT parasite on HCT-8 cell corresponding to Fig 5E with no annotation.
(TIF)

S1 Table Pull down results. (XLSX)

S2 Table List of primers. (XLSX)

S3 Table Dilution of antibodies and dyes. (XLSX)

S4 Table Raw data. (XLSX)
